# Erosion or normal variant? 4-year MRI follow-up of the wrists in healthy children

**DOI:** 10.1007/s00247-015-3494-6

**Published:** 2015-12-04

**Authors:** Derk F. M. Avenarius, Lil-Sofie Ording Müller, Karen Rosendahl

**Affiliations:** Faculty of Health Sciences, University of Tromsø, 9037 Tromsø, Norway; Department of Radiology, University Hospital of North Norway, Tromsø, Norway; Department for Radiology and Intervention, Oslo University Hospital, Oslo, Norway; Department of Radiology, Haukeland University Hospital, Bergen, Norway; Department of Clinical Medicine K1, University of Bergen, Bergen, Norway

**Keywords:** Cartilage, Children, Erosion, Magnetic resonance imaging, Normal variants, Wrist

## Abstract

**Background:**

A large proportion of healthy children have wrist changes on MRI, namely carpal depressions, findings that have been described as pathological in children with juvenile idiopathic arthritis.

**Objective:**

We performed follow-up imaging in a cohort of healthy children to evaluate carpal surface depressions over time, focusing on the presence of overlying cartilage as a potential discriminator between normal variants and true erosions.

**Materials and methods:**

74 of the initial cohort of 89 healthy children (83%) had a re-scan of their wrists using the same protocol, including coronal T1 and fat-saturated T2 sequences. A cartilage-selective sequence was added for this study. We registered number and location of bony depressions and presence of overlying cartilage.

**Results:**

The total number of carpal depressions increased by age group and over time; their location was unchanged in 370 of 487 (76%) carpal sites and 91 of 117 (78%) metacarpal sites. In total, 426 of the 1,087 (39.2%) bony depressions were covered by cartilage, with a decreasing percentage by age (*P* = 0.001).

**Conclusion:**

Normal appearances during growth, such as bony depressions, should not be mistaken for pathology. There must be additional findings to support a diagnosis of disease. A cartilage sequence may add to the diagnostic image analysis.

## Introduction

Juvenile idiopathic arthritis is a heterogeneous condition that includes all forms of chronic arthritis of unknown origin with onset before 16 years of age. It is characterised by chronic synovial inflammation with a potential risk of progressive joint damage and serious functional disability [1–4]. Juvenile idiopathic arthritis affects 1–2 in 1,000 children [5–8]. There is increasing evidence that many, if not most, children with juvenile idiopathic arthritis have ongoing disease into adulthood, particularly if they are HLA-B27-positive [9]. Moreover, research has shown there might be a therapeutic window of opportunity during early disease and that early initiation of therapy is associated with slower progression of joint damage and higher rates of remission [10]. This highlights the need for more sensitive disease markers because many of the existing markers, based on clinical, laboratory and conventional imaging, are imprecise and inaccurate.

Joint damage evaluation in juvenile idiopathic arthritis has traditionally been performed by radiographic scoring methods; these methods, however, are not sensitive, particularly for disease in early stages [11–14]. MRI, on the other hand, can be used to image synovitis and bone oedema/inflammation as well as damage to cartilage and bone, and is believed to detect erosive changes with greater sensitivity than radiography, particularly in early disease [15]. The value of MRI as an advanced method to evaluate disease activity and disease damage in adults with rheumatoid arthritis is under active investigation by a research consortium called Outcome Measures in Rheumatology Clinical Trials (OMERACT) [16, 17]. However, the results from OMERACT studies are not directly applicable to children, because adult rheumatoid arthritis is different from JIA and because the growing skeleton of a child has different appearances on imaging and reacts differently to disease processes than does the mature skeleton. This has fueled paediatric imaging groups, such as the Health-e-Child Radiology Group and the OMERACT special interest group “MRI in Juvenile Idiopathic Arthritis” to join efforts; however no internationally validated and accepted scoring system for MRI in juvenile idiopathic arthritis exists for any joint [18].

We have previously shown that on MRI, a large proportion of healthy children have changes similar to those described in children with juvenile idiopathic arthritis, namely carpal depressions, bone-oedema-like changes and joint fluid ≥2 mm [19–21]. To further characterise normal growth of the wrist we performed a 4-year follow-up of this cohort and included a cartilage-specific sequence to examine the relationship between bony depressions and cartilage coverage.

## Materials and methods

The initial Tromsø wrist cohort included 89 healthy children ages 6–15 years and residing in Tromsø, Norway. After an interval of 4 years, the children and caregivers were approached a second time by postal mail and invited for a follow-up MRI and radiograph of the left wrist. The results of the radiographs will be published separately. Seventy-four (83%) children, now ages 10–19 years, accepted the invitation and 64 were scanned in the summer and early autumn. On arrival to the radiology department, all children were (1) checked for contraindications to MRI, (2) asked whether they had sustained recent injuries to the left wrist, e.g., within the last 2–3 weeks and (3) asked whether they participated in organised sports or training, and if so, the number of training sessions per week.

The MR examinations were again performed on a 1.5-T MR scanner (Intera 2.3; Philips Healthcare, Best, the Netherlands) with master gradients and a four-element wrist coil. All the children were awake, with no sedation during the scan. The scans were performed in a supine position with the wrist alongside the body. Sequence parameters were as follows: (1) coronal T1-W fast spin-echo, repetition time (TR)/echo time (TE) 561/6.8 ms, 3 echoes and number of signals averaging 6, with 40 slices in 3 stacks, slice thickness 0.9 mm, acquired voxel size 0.69 × 0.72 × 0.9 mm, reconstructed voxel size 0.25 × 0.25 × 0.9 mm, with parallel imaging with a reduction factor of 1.6, scan time 4 min 11 s; (2) coronal T2-W fast spin-echo, TR/TE 3,165/70, 10 echoes, fat-suppressed using spectral selection attenuated inversion recovery, number of signals averaging 4, 14 2.5-mm slices, acquired voxel size 0.31 × 0.40 × 2.5 mm, reconstructed voxel size 0.15 × 0.15 × 2.5 mm, scan time 3 min 56 s. A third sequence, as follows, was added to the original protocol as the first sequence: coronal 3-D water-selective steady-state spoiled gradient cartilage series with 60 × 0.75-mm slices, acquired voxel size 0.38 × 0.38 × 1.5 mm, scan time 4 min 7 s. The T1-W and water-selective-cartilage series were automatically reconstructed in the two additional perpendicular planes. The study was approved by the Regional Ethics Committee of Northern Norway (reference number 2013/385/REK North).

### Image analysis

**Fig. 1:**
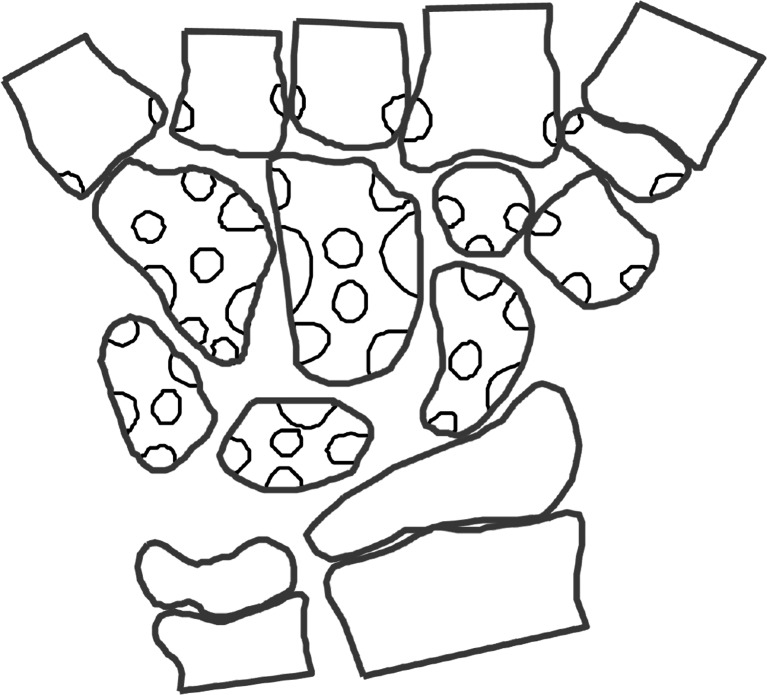
Template used to score the bony depressions by location. Areas in the middle of the bones refer to volar or dorsal sides of the bones. The different locations for each bone were grouped for the statistical analysis (Table 1 and 2)

The wrist MRIs were analysed in consensus by three radiologists with a special interest in paediatric musculoskeletal radiology (K.R., 15 years of experience; L.S.O.M., 9 years of experience; D.A., 12 years of experience), using the hospital picture archiving and communication system. The number and location of bony depressions, defined by a bony indentation, were assessed from the coronal views (K.R., D.A.), confirmed in an additional reconstructed plane as appropriate and registered on a specific template (Fig. 1). The template was derived from the templates used in our previous study, reflecting the typical locations of bony depressions in the normal cohort [20], with one modification, i.e. reducing from 4 to 3 sites on the trapezium (this affected only one depression that was relocated to the nearest position on the template). We noted the presence of cartilage overlying each of the depressions (yes/no) as well as the preferred image plane to determine the cartilage coverage. On a second assessment, the two examinations from 2009 and 2013 were compared side by side, and each of the depressions seen in 2009 was scored as unchanged or disappeared. Depressions seen only in 2013 were scored as new.

### Statistical analysis

The children were grouped according to chronological age, similar to the grouping used in 2009, for direct comparison. The follow-up examination was performed with an interval of 3.7–4.8 years (mean 4.2, standard deviation [SD] 0.2) from the initial exam. Group I was now 10–12 years (mean 11.4), group II was 12–14 years (mean 13.1), group III was 14–16 years (mean 15.2) and group IV was 16–19 years (mean 17.3). A chi-squared test was used to examine possible associations between gender and number of bony depressions. We performed a one-way between-group analysis of variance (independent variable age, dependent variable number of bony depressions) to examine the impact of age on the number of bony depressions and on the rates of depressions covered with cartilage (dichotomised). The analyses were performed using Statistical Package for the Social Sciences, v. 21 (IBM, Armonk, NY). Alpha <0.05 was used to define statistically significant differences.

## Results

Seventy-four (35 males) of the original 89 (45 males) healthy volunteers (83.2%) were included in the follow-up study. None of the children had sustained recent injury to the wrist, and all remained healthy with no signs of inflammatory joint disease. All 74 re-examinations held acceptable technical quality, as opposed to 84 (44 males) on the initial MRI, leaving 71 children (33 males) with two acceptable MR scans for longitudinal analysis. All 74 follow-up scans were used for assessment of cartilage. Eight of the children/adolescents (4 males) were left-handed. The number of organised training sessions in addition to the normal school gymnastics varied from none to 10 weekly (mean 3.4, SD 2.1). No differences in the occurrence of carpal depressions were found between males and females, and the data were therefore pooled for further analysis.

### Bony depressions

The mean number of total carpal depressions increased with increasing age, from 11.0 in group I to 15.0 in group IV (*P* < 0.001, Table 1). Except for the hamate, this was also the case for each bone separately, peaking at 14–16 years of age (Table 1). The total mean number of metacarpal depressions increased from 2.8 in the youngest age group to 3.8 in the older age group (*P* = 0.06) (Table 1).

**Table 1:** Carpal depressions in 71 healthy children and adolescents as seen on a T1-weighted fast spin-echo sequence, presented as mean (standard deviation)

	Group I	Group II	Group III	Group IV	*P*-value*
	10–12 years (*n* = 19)	12–14 years (*n* = 15)	14–16 years (*n* = 17)	16–19 years (*n* = 20)	
Total	11.0 (2.9)	14.0 (2.7)	15.0 (2.1)	15.0 (3.5)	<0.000
Trapezium/oid	0.4 (0.5)	0.5 (0.5)	1.1 (1.0)	0.6 (0.6)	0.01
Scaphoid	1.2 (0.8)	1.2 (0.7)	2.5 (0.9)	1.8 (0.8)	0.04
Lunate	1.3 (0.8)	2.7 (0.5)	2.5 (0.9)	2.4 (1.0)	<0.001
Capitate	2.8 (0.7)	3.6 (0.9)	3.5 (0.9)	2.9 (0.9)	0.02
Hamate	1.2 (1.1)	1.8 (1.2)	1.6 (0.5)	1.6 (0.5)	0.2
Triquetrum	1.2 (0.6)	1.3 (0.9)	1.6 (0.9)	2.2 (0.9)	0.006
Metacarpal	2.8 (1.2)	2.7 (1.5)	3.1 (1.1)	3.8 (1.6)	0.06

*P-values from one-way between-group analysis of variance

**Fig. 2:**
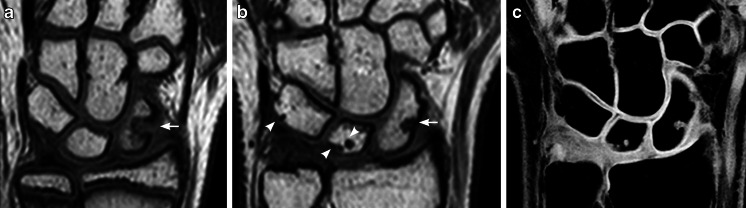
Proximal carpal row. **a**, **b** Coronal T1-weighted turbo spin-echo MR image in a 13-year-old boy (**a**) shows a large bony depression in the scaphoid bone (*arrow*). A 4-year follow-up (**b**) the depression is still seen but is less pronounced. New bony depressions are seen on the surface of the lunate and triquetral bones (*arrowheads*). **c** Coronal water-selective (coronal volumetric steady-state spoiled gradient echo) cartilage sequence shows that both old and new depressions are covered with cartilage

**Fig. 3:**
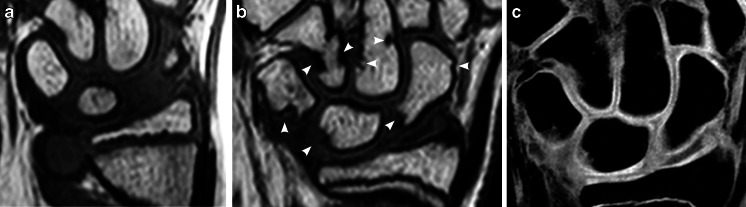
Carpal bones. **a**, **b** Coronal T1-weighted turbo spin-echo MRI in a 12-year-old boy (**a**) shows smooth carpal bones. Four-year follow-up MRI (**b**) shows several new bony depressions in almost all carpal bones (*arrowheads*). **c** Coronal water-selective (coronal volumetric steady-state spoiled gradient echo) cartilage sequence shows that all carpal depressions are covered by cartilage at follow-up

**Fig. 4:**
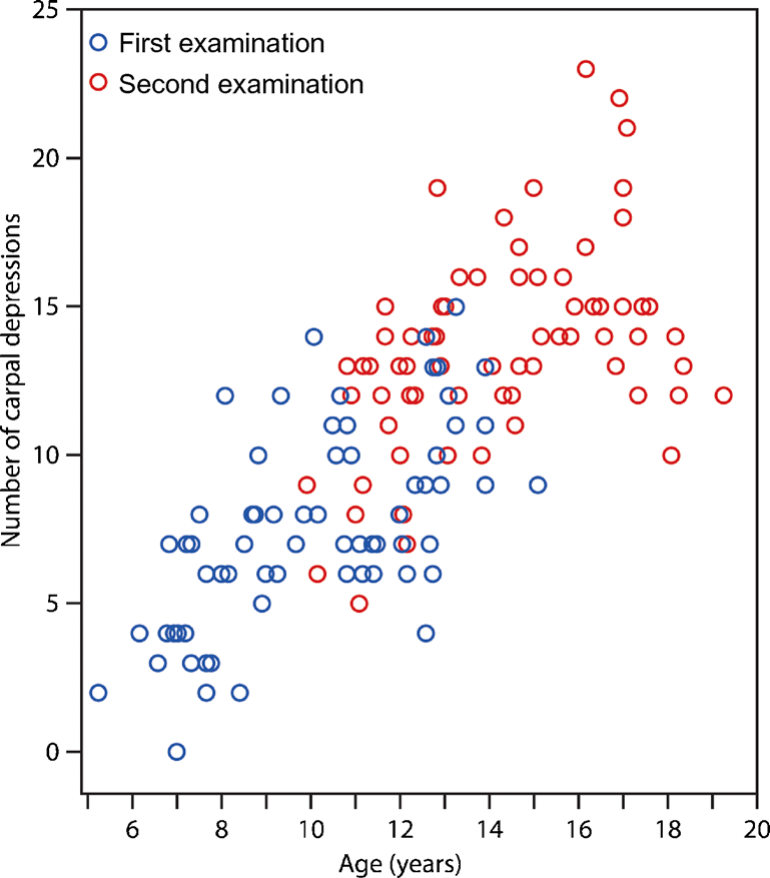
Bony depressions on first vs. follow-up (second) examinations. Scatter plot shows the total number of bony depressions by age of the first examination (*blue*) and second examination after a mean of 4.2 years (*red*)

The total number of carpal depressions increased over time (Table 1), with a total mean of 7.7 (range 0–15) in 2009 vs. 13.6 (range 5–23) in 2013 (Figs. 2, 3 and 4). Their location was unchanged in 370 out of 487 (76%) carpal sites and in 91 out of 117 (78%) metacarpal sites. Twenty-six of 117 (22%) metacarpal depressions and 117 of 370 (32%) carpal depressions had disappeared.

### Cartilage

**Fig. 5:**
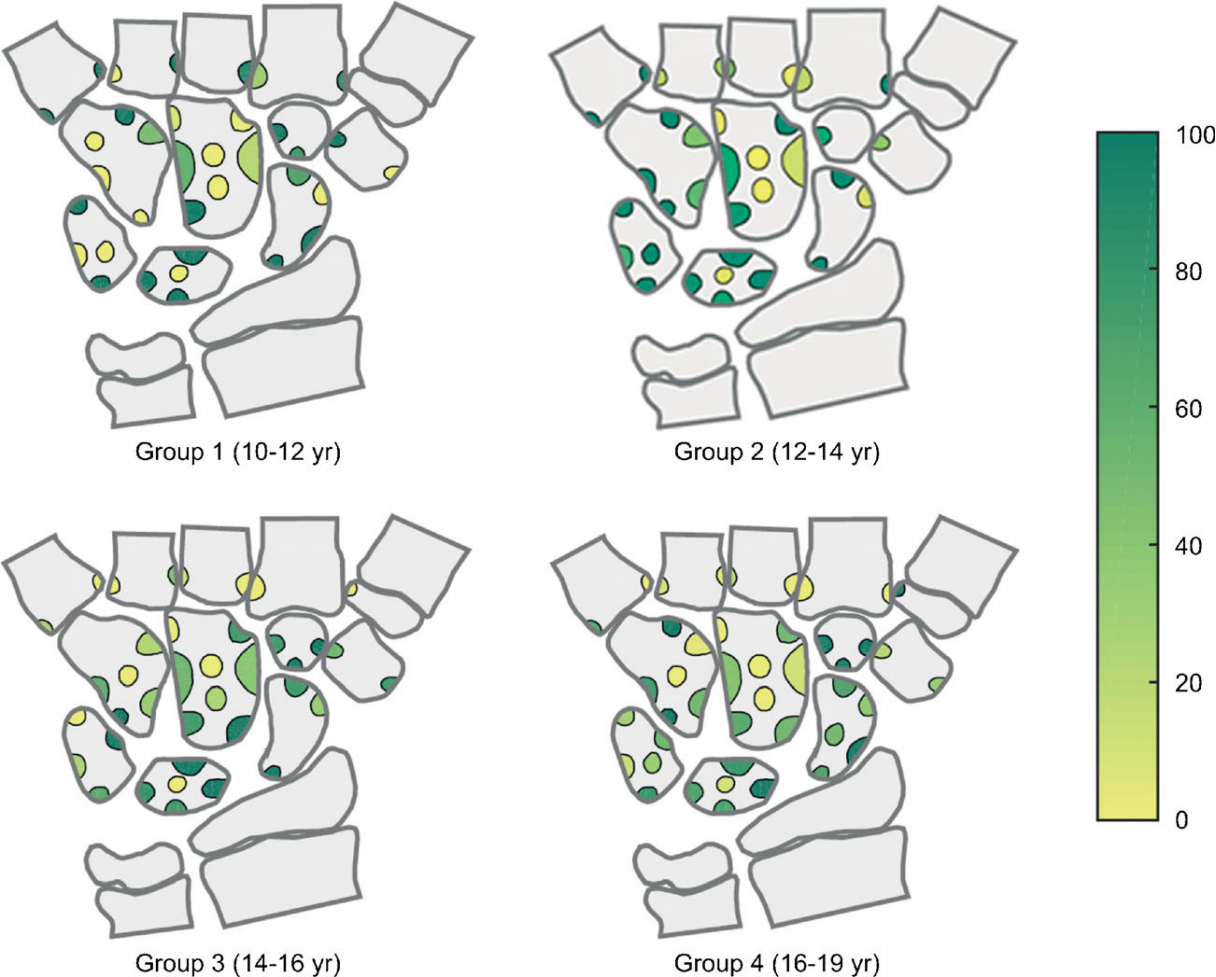
Percentage of depressions covered with cartilage (*yellow* 0%, *green* 100%), organised by age group and location within each bone. Central depressions, i.e. volar or dorsally located, were identified on the sagittal and axial reconstructions and were often scored as not covered. There is a much higher percentage of covering on the articular surface of the carpal bones in all age groups. There is a high percentage of covering on the base of the metacarpals in the youngest group

**Fig. 6:**
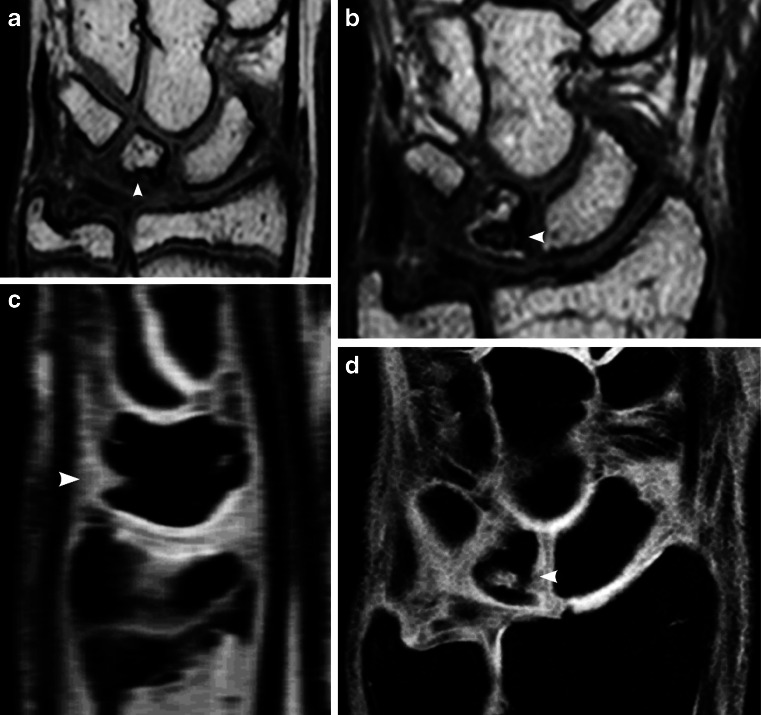
Depressions (*arrowhead*) in the lunate bone. **a**, **b** Coronal T1-weighted turbo spin-echo MRI in a 14-year-old boy (**a**). At 4-year follow-up MRI (**b**) the depression appears more irregular. **c**, **d** Coronal water-selective (coronal volumetric steady-state spoiled gradient echo) cartilage sequence at 4-year follow-up. Sagittal reconstruction (**c**) and coronal image (**d**) show intermediate signal on the articular side of the depression, which was therefore classified as not covered. The sagittal reconstruction (**c**) locates this depression to the dorsal side of the lunate

**Fig. 7:**
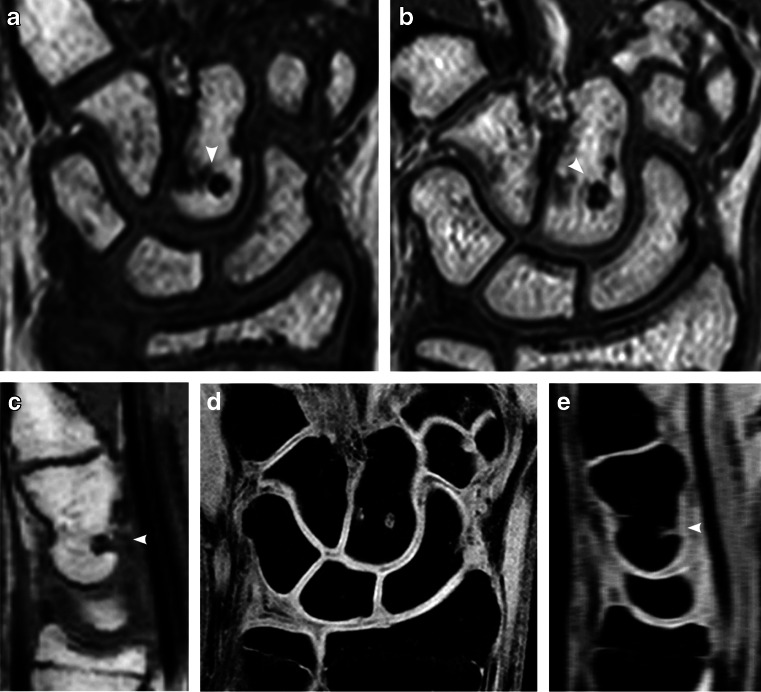
Depression (*arrowheads*) on the volar side of the capitate bone. **a**–**c** T1-weighted turbo spin-echo MRI in a coronal plane in an 11-year-old boy (**a**) and unchanged at 4-year follow-up seen on coronal image (**b**) and, better, in a sagittal reconstruction (**c**). **d**, **e** On the water-selective (volumetric steady-state spoiled gradient echo) cartilage sequence at 4-year follow-up, the signal within and covering the depression is lower than that of the articular cartilage, as seen in a coronal image (**d**) and, better, in a sagittal reconstruction (**e**), suggesting it is not covered

**Fig. 8:**
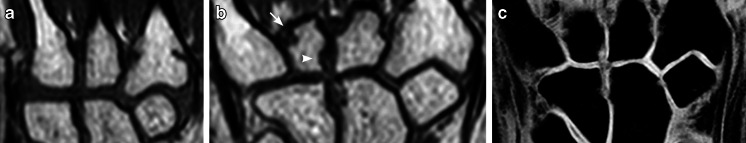
Base of the fourth metacarpal bone. **a**, **b** Coronal T1-weighted turbo spin-echo MRI demonstrates a smooth outline in a 10-year-old girl (**a**); however two depressions, one proximal (*arrowhead*) and one distal (*arrow*), are seen at 4-year follow-up (**b**). **c** Coronal water-selective (volumetric steady-state spoiled gradient echo) cartilage sequence at 4-year follow-up shows that the proximal depression is covered but the distal is not

In total, 426 of the 1,087 (39.2%) bony depressions were covered by cartilage as assessed on the water-selective-cartilage sequence, with a decreasing percentage by age group (*P* = 0.001) (Table 2, Figs. 5, 6, 7 and 8). The rates of cartilage-covered bony depressions varied according to location (Table 2, Fig. 5).

**Table 2:** Depressions judged to be covered by cartilage on a water-selective cartilage MR sequence, presented by location and age group as percentage (standard deviation) of depressions

	Group I	Group II	Group III	Group IV	*P*-value*
	10–12 years (*n* = 18)	12–14 years (*n* = 16)	14–16 years (*n* = 19)	16–19 years (*n* = 21)	
Total	0.52 (0.2)	0.46 (0.2)	0.38 (0.1)	0.32 (0.1)	0.001
Trapezium/oid	0.86 (0.4)	0.50 (0.5)	0.79 (0.3)	0.54 (0.5)	0.2
Scaphoid	0.25 (0.3)	0.34 (0.4)	0.44 (0.5)	0.50 (0.4)	0.3
Lunate	0.62 (0.4)	0.62 (0.2)	0.57 (0.3)	0.53 (0.4)	0.8
Capitate	0.42 (0.3)	0.33 (0.2)	0.35 (0.2)	0.15 (0.2)	0.004
Hamate	0.46 (0.5)	0.58 (0.4)	0.34 (0.4)	0.31 (0.4)	0.2
Triquetrum	0.81 (0.3)	0.82 (0.2)	0.52 (0.4)	0.52 (0.3)	0.01
Metacarpal	0.50 (0.4)	0.17 (0.2)	0.058 (0.1)	0.067 (0.1)	<0.000

*One-way between-group analysis of variance

Of the 661 bony depressions where cartilage-coverage could not be seen, 177 (27%) were located at the insertion of the inter-metacarpal ligaments, 127 (19%) at the insertion of the capitate–hamate ligament, 91 (14%) at the insertion of the radio–ulnar collateral ligaments, 58 (9%) between the capitate and trapezoid bone, and 56 (8%) at the volar and dorsal attachments of radial and ulnar carpal ligaments (Figs. 6, 7 and 8). The remaining 152 depressions were located in other juxta-articular areas.

Ninety-two percent (1,001 of 1,087) of the bony depressions were best evaluated on the coronal scan.

## Discussion

We have shown in a group of healthy children that most carpal depressions are stationary and that new depressions appear with skeletal maturation. Amongst 10- to 19-year-olds, 40% of the bony depressions were covered by cartilage whilst nearly half of those considered uncovered were located at or near the attachments of the main carpal ligaments. The remainder represents other carpal ligament attachments, vascular channels, and areas that are difficult to assess.

Carpal bones form through enchondral ossification from a single ossification center, in a counter-clockwise direction (anatomical position), starting with the capitate. Each bone has 4–7 tight articulations with adjacent bones and is tied together with numerous ligaments. The majority of erosions in children with juvenile idiopathic arthritis have been reported at these articulations [20, 22].

Increasing numbers of bony depressions were seen over time, particularly at the bases of the metacarpals and in the triquetrum, scaphoid, capitate and lunate bones, all representing areas of significant growth during the study period of 4 years (Fig. 3). Moreover, the number of depressions in several of the carpal bones, namely the trapezium, the trapezoid, the scaphoid and the capitate, seemed to peak just before reaching maturity. One explanation of this peak may be that the additional bone surface irregularities, seen on T1 series, occur from normal but irregular enchondral bone maturation (Fig. 2), which, unlike ligament attachment, does not contribute to the final shape of the bone. The oldest volunteers in our cohort still had many bony depressions at follow-up, and it is therefore expected that the adult skeleton also has these features.

In a previous paper we demonstrated that carpal depressions in healthy children may resemble those seen in children with known juvenile idiopathic arthritis [20], thus hampering the diagnosis of permanent bone damage. Under the presumption that damage to or thinning of the cartilage precedes destructive bone change, we hypothesised that bony irregularities seen in healthy wrists are most likely covered with articular cartilage unless located at or near ligamentous attachments or vascular channels. Currently, several sequences, both anatomical and structural, are available for cartilage imaging [23–26]. For this study we considered proton-density sequences with and without fat saturation, T2-W and water-selective-cartilage sequences, all of which have their pros and cons. The proton-density sequence without fat saturation gives good anatomical detail and high contrast to joint fluid at the cost of slice thickness; adding a fat-saturation pulse increases the contrast between cartilage and subchondral bone at the cost of the signal-to-noise ratio, whilst T2 provides typical dark cartilage with high contrast to fluid at the expense of cartilage signal. We chose a water-selective steady-state spoiled gradient 3-D sequence because of its high contrast among cartilage, bone and joint fluid, its time efficiency and its facilitation of multiplanar reconstruction, which allows for detailed comparisons with the findings from the T1-weighted images.

The brim of cartilage covering the bony depressions varied, not only with age but also between separate bones and within the same bone (Fig. 5). This variation within and between bones could be in part a result of methodological difficulties — articular surfaces were easier to assess, because of higher cartilage signal, than surfaces located at the dorsal and volar aspects of the wrist (Figs. 6 and 7).

**Fig. 9:**
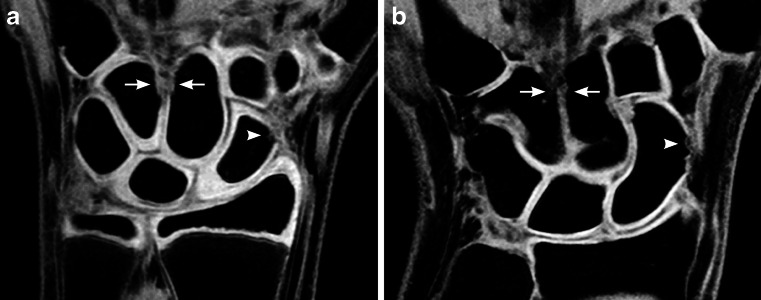
Variable cartilage thickness. **a**, **b** Coronal water-selective (volumetric steady-state spoiled gradient echo) cartilage sequence in an 11-year-old boy (**a**) and an adult (**b**). Little, if any, cartilage is visible at the sites of the intracarpal ligament between the hamate and capitate bones (*arrows*) and the radial border of the scaphoid bones (*arrowhead*)

The cartilage sequence was particularly helpful in the youngest children because of their relatively thick cartilage (Fig. 9). Cartilage imaging could be helpful to distinguish normal variants from true erosions in the carpal bones, particularly when they occur in the articular surface, where erosions frequently occur in juvenile idiopathic arthritis [20, 22]. In the proximal metacarpals cartilage covering might have a role in diagnosing true erosions only in the younger children because cartilage coverage in this area was rarely seen in the older age groups. The addition of a cartilage sequence to a standard protocol could be useful in clinical practice, but further research is needed to compare cartilage coverage in healthy children and in children with wrist-involved juvenile idiopathic arthritis.

The assessment of bony depressions at sites of ligament attachments and vascular channels was not eased by the additional cartilage sequence and indeed required detailed anatomical knowledge. Even in our youngest individuals it was difficult to see cartilage signal at some of these locations (Fig. 9).

One of the limitations to our study was the subjective assessment and consensus reading; however objective measurements of small, irregular structures are flawed with methodological difficulties. A second limitation is that the healthy cohort was entirely Caucasian and recruited from one particular city, and with the exception of 10 individuals, all were scanned during summer or autumn. A third limitation is that the MR scans were performed on a 1.5-tesla system with relatively low resolution compared to what could be archived with a 3-tesla system. Last, enhanced images could not be obtained, because this would be unethical, thus leaving the role of contrast enhancement in the differentiation between true erosions and growth-related bony depressions unanswered. In opposition to what has been reported for true erosions in active disease [22], one might speculate that growth-related bony depressions are not associated with increased enhancement of surrounding tissues. However, in cases of inactive disease, contrast enhancement most likely plays a less important role, underscoring the importance of an additional cartilage sequence.

## Conclusion

MRI appearances of wrists in healthy children vary over time. Normal growth changes such as bony depressions should not be considered as signs of juvenile idiopathic arthritis pathology unless there are additional findings to support disease. A cartilage sequence might add to the diagnostic image analysis in that nearly half of the observed bony depressions are covered with cartilage. It is reasonable to believe that true erosions are not covered by cartilage, based on the presumption that cartilage destruction occurs before bony destruction; however, this remains to be verified.
